# Generic formulations: availability and applicability for exposure assessment

**DOI:** 10.1038/s41370-025-00837-4

**Published:** 2026-03-16

**Authors:** Rosemary T. Zaleski, Andreas Ahrens, Richard A. Becker, Kristin Isaacs, Katherine Phillips

**Affiliations:** 1 Lumina Consulting L.L.C., Hillsborough, NJ USA; 2 Institute for Environmental Strategies (Oekopol), Hamburg, Germany; 3 American Chemistry Council Long-Range Research Initiative (ACC LRI), Washington, DC USA; 4 United States Environmental Protection Agency, Office of Research and Development, Research Triangle Park, NC USA

**Keywords:** Chemical in products, Computational exposure science, Exposure assessment, Workplace exposures, Exposure modeling, Sustainable development, Risk assessment

## Abstract

**Background:**

Generic formulations (GFs), representations of the composition of consumer and industrial products based upon function rather than specific substances, can be useful tools for exposure and risk assessors. They provide a way to share the information needed for screening assessments of product safety without disclosing specific product formulations. GFs provide a structure for organizing the product/process type and associated qualitative and quantitative chemical ingredient information.

**Objective:**

This perspective seeks to explain and highlight how GFs can be useful in exposure and risk evaluation, provides examples of GF information sources, and offers suggestions for further development of GFs. The primary emphasis here is on available information for industrial products, as this space is historically data-poor with respect to publicly available GF information.

**Methods:**

Publicly accessible GF references were located and mapped to the Organization for Economic Co-operation and Development (OECD) harmonized use categories. Authors’ personal knowledge of sources of GFs played a key role because a great deal of GF-related information is embedded within spreadsheets or documents posted on the European Chemical Agency (ECHA) website or websites of commercial enterprises, and these sources may not be readily identified using typical internet or literature search strategies.

**Results:**

Industrial GF information was found for 13 of 20 OECD use categories, but only for 20 of 91 OECD product-subcategory combinations. Limitations in findability and disparity in availability across sources support the value of developing common, consistent practices and platforms across stakeholder communities for sharing GFs and other exposure-relevant information.

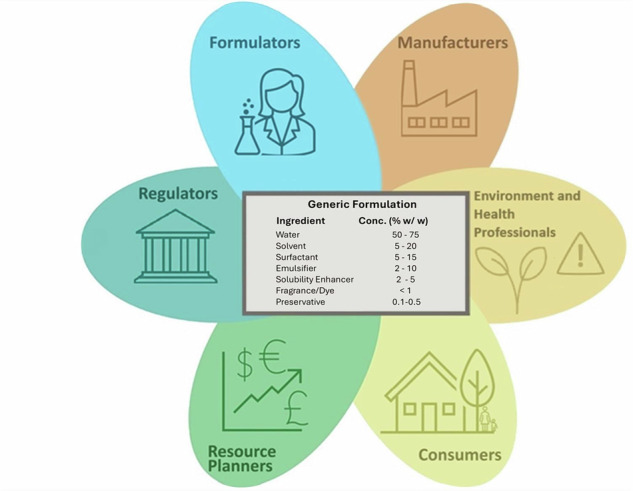

**Significance:**

This paper highlights available data, makes the case for systematic development and public availability of GF data, and explains the benefits of having this information available for various user sectors.

## Introduction

Robust assessment of chemical exposure and risk depends upon accurate chemical use information, including: an understanding of product or process types in which a substance is utilized; the function a substance may have in certain products, materials or processes and the concentration at which this function is delivered; the identification of the end-user population; and the estimation of substance mass-flows potentially released into outdoor and indoor environments. For formulated products in commerce, the publicly available data to address these elements may be limited, largely due to laws and regulations that protect proprietary formulations [1]. Chemical use information is also critical for product stewardship, chemical regulation, and emerging sustainability initiatives. Without such information, regulators and chemical suppliers in the upper part of the supply chain usually have difficulties in setting appropriate priorities for risk management.

Several recent scientific activities have addressed the need for better characterization of chemical use. Non-Targeted Analysis (NTA) is being used to identify constituents and/or constituent structures; these studies can detect hundreds of structures in a given sample [2–5]. Development and expansion of Quantitative Structure Use Relationships (QSURs), which predict chemical function from chemical structure, can be used to estimate potential chemical presence and, in some cases, relative amounts of chemicals in a product type [6, 7]. Development of Free Accessible Interoperable and Reusable (FAIR) databases containing and organizing chemical use information is making data more accessible [8, 9]; while mining of commercial sales data for information on uses of chemicals has identified use trends and regional variations [10].

Generic formulations (GFs) are generalizations of specific products (in terms of ingredient types and their concentrations) that are utilized in exposure and risk assessments in place of specific individual formulations. These formulations are often chemical-agnostic (i.e., they do not specify specific chemicals in a product) and focus on chemical functions of components needed for a product to work in a specific way (Table 1). GFs have applications that span development, manufacturing, regulatory, risk assessment, and consumer activities.

**Table 1:** Types and examples of Generic Formulation information. Generic formulations may have varying levels of information, and these examples illustrate how these levels are categorized within this paper. Denoted as G = full generic formulation—includes all or key ingredients and concentration information. pG = Partial Generic Formulation- includes some ingredient or concentration information.

Type	Example	Full or Partial
Basic, typical or representative formulation. Based upon ref. [40]. Comment: Full formulation (100% of components) specified.	Wood polish, cream type Mineral oil, 55% Emulsifying agent, 3% Isopropanol, 4% Water, 38%	G
Frame formulation (a type of generic formulation based upon specific regulatory requirements) of a cosmetic product. Based upon ref. [41]. Comments: Maximum concentrations provided; if these concentrations were used for each component, mass balance would be exceeded.	Skin care cream, lotion, gel Ingredient Max conc %w/w Oils, waxes and fats 30 Silicones incl. volatile silicones 20 Humectants 20 Thickener 12 Ethanol 10 Additional ingredients 10 Bulking agents 5 UV filters 5 Emulsifying agents, surfactants 5 Preservatives, antimicrobials 2 Colorants 2 Parfum 1 Aqua to 100	G
Ranges of composition. Based upon ref. [42]. Comment: Although not the case for this specific example, using the upper bound of the range for each constituent may exceed mass balance in some cases.	Liquid oven cleaners: Anionic surfactants, 0–10% Solvents, 0–10% Additives: Monoethanolamine, 0–5% Potassium carbonate, 0–10% Sodium metasilicate, 0–5% Sodium hydroxide, 0–0.5% Water: Up to 100%	G
Standard or well-known components. Based upon ref. [40]. Comment: Informs likely presence in a product but does not support more precise quantification.	Modeling Clay: Kaolin, gypsum, petrolatum, wax, castor oil, vegetable dye	pG
Weight fractions of a given substance within various products. Based upon ref [14]. Comment: Informs likely presence and quantification for the specific constituent, but no additional information on the formulation.	H_2_O_2_ (weight%) in household: Machine washing detergent: 0 Laundry additives, liquid bleach/gel: 0–8.5 Surface cleaners: 0–5 Toilet cleaner: 4.6	pG

This perspective discusses the value of GFs for filling knowledge gaps in use information and summarizes available resources for GFs or information to assist in their development, with a focus on industrial products (a typically data-poor sector with respect to exposure assessment). Specific product categories for which additional data development may be useful are identified. Finally, activities that would increase the availability and accessibility of chemical data for developing GFs are proposed. This paper highlights available data, advocates for a more formalized framework to organize and obtain these data, and explains the benefits of having this information available for various user sectors.

### Definitions for GFs

GF information may be shared at various levels of detail (Table 1). The most complete GFs are intended to provide a basic or typical representation of a product’s composition. Components may be listed as ingredient functions or specific (group of) substances, or by a combination of both function and specific (group of) constituents. The amount of each ingredient component (substance or function) may be provided as a maximum concentration or weight fraction within the formulated product or as a concentration range. GFs are advantageous because they can cover multiple brand-name products that fall within a single product category, and they can make information available while protecting proprietary formulations. Less complete but still relevant GF information may list only standard or well-known components, with or without concentrations. For the purposes of this document, we consider all levels of relevant information as GFs, with the latter case being referred to as partial generic formulations (pGFs). Here, we consider a GF to include three separate information elements:

- Product type for which the general formulation is representative (e.g., machinery paint, firefighting agents, etc.)
- Functions of substances/chemistries within the product type (e.g., surfactant, biocide, fragrance, cross-linker, etc.) or specific constituents of the product
- Point, distribution, or range of concentration levels associated with one or more of the expected function or listed constituent(s)

### The value of GFs

**Fig. 1:**
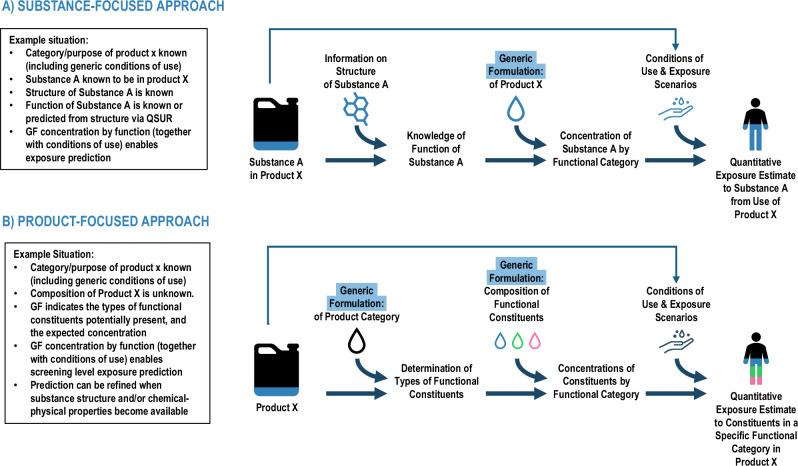
Example applications of GFs in exposure assessment. Two approaches are presented: substance specific (**A**), and product specific (**B**). Steps show how GF information may be used to develop quantitative exposure estimates. Exposure quantification capabilities will depend both upon the level of information presented in the GF and its application—a maximum weight fraction may be used as an upper bound screening level, a central tendency or typical weight fraction may be used, or Monte Carlo simulations can be run when a weight fraction range is specified.

Multiple use-related considerations go into performing a chemical exposure assessment, including likelihood of presence in a product, concentration in the product if present, end-user populations and handling practices, and emissions throughout the product and chemical lifecycle (further discussion in Supplementary Information SI-1). GFs can play a key role in helping to fill gaps in understanding the likelihood of chemical presence in formulated products and the concentration if present (Fig. 1, example in SI-2); one such example is the integration of GFs with QSUR models. QSURs predict function from chemical structure. Refined models could be developed that address the prediction of these within specific categories or sectors of products. Since GFs most often contain information in terms of function, predicted functions from QSURs can be used in concert with GFs to identify defensible screening-level concentrations (i.e., those appropriate for lower-tier analyses) in different products for specific chemicals with no known data. This essentially allows for estimating presence and concentration within a product type based on known or QSUR-predicted function. These approaches are appropriate for use in high-throughput exposure estimation activities such as chemical prioritization. Conversely, GFs could provide information for evaluating predictions of concentration ranges for function-product category pairs returned by new QSURs.

Examples of regulatory applications of GFs include the generic (“frame”) formulations defined in the EU Biocidal Products Directive and EU Regulation 1223/2009 on Cosmetic Products [11, 12]. These regulations include specific guidance for frame formulation development. Within this regulatory context, frame formulations are applied to facilitate assessment of product safety, and requirements and/or fees for substances/formulations falling within the frame formulations may be reduced. Generic formulation information (constituents/constituent classes) has also been used to support the development of voluntary standards and safe handling practices that support occupational and consumer safety [13].

Also, less specific or less complete formulation information (pGF information), such as the maximum or range of weight fraction for a substance or chemical category, can be useful and may be provided across multiple product categories (such as found in [14, 15]). This information enables quantitative assessment of the substance/chemical category being addressed using conservative estimates of concentration. Examples of quantitative assessments employing this approach are found on the HERA website and in Safford et al. [16, 17].

This paper explores the availability of GFs for industrial use in mixtures and materials. The focus of this work is on industrial formulations, since previous work has identified multiple resources for consumer products [1]. Consumer information observed while collating industrial information was also included, however.

## Methods

### Compilation of data resources informing GFs

Previous work identified sources for GFs as part of supporting information for a QSUR Summit [1]. These GFs were largely consumer-based. During the Summit it was identified that participants were aware of additional GF information for industrial sources that were unlikely to be found via literature search because they were not journal or book publications- i.e., the European Chemical Agency (ECHA) Use Map website [18], the Organization for Economic Co-operation and Development Emission Scenario Documents (OECD ESDs) [19], and other relevant websites or references known to the authors. The main purpose here was to assess if resources known to the authors could expand knowledge of substance use in the industrial and/or professional sector and thereby complement work already identified and being applied in the consumer area.

An in-depth literature review using a structured search strategy was not conducted for the purpose of this exercise. Rather, this was a review of selected sources and not an exhaustive systematic review. Historic sources predating 1985 were not included in the analysis of information availability, as their representativeness of current product composition was unknown. Information sources known in advance to be for specific individual products (e.g., Safety Data Sheets—SI-3, pesticide registrations) were not included. Information from all references was treated equally—no weighting was applied. This effort focused on the availability of GFs and did not assess the methods used for GF development, which could include measurement, recipe knowledge, expert elicitation, etc.

### Organization of GF information

Identified GF information was assigned to OECD harmonized product categories [20] to systematically document product information availability and assist with identifying future areas for focused information gathering (note, the product categories and subcategories in the 2025 edition cited here remain the same as those in the 2017 edition available at the onset of this activity). The OECD harmonized use categories are divided into products and articles (definitions provided in SI-3). There are 19 product categories, including a category of “other.” Each product category can have subcategories, yielding a total of 91 product-subcategory combinations. Article categories are based upon 9 material types and associated categories, for a total of 69 material-category combinations.

This document does not target article composition in a substantive manner. It includes information for plastics, as documents supporting plastics formulation (master-batching and compounding) were available on the ECHA website, and based upon the author’s judgement, it was felt that materials that go into the production of mass polymers would be captured in this information. For other article types that have water-based processes in which composition splits between what goes into wastewater and what remains associated with the article (e.g., paper and textile articles) or special products based upon reactive resins, however, the use of formulation information may be more challenging to apply. An additional and expanded effort to more carefully examine articles would need to include different information sources, and is beyond the scope of this article.

For each category, GF information was categorized as either applying to consumer or industrial use. Consumer use was defined as widespread use in residential settings. Industrial use included manufacturing or formulating under industrial settings. It should be noted that parsing information into consumer or industrial settings requires some interpretation. For example, “industrial” includes manufacture, formulation or use of products in industrial settings, as well as professional or institutional product use. If data were provided for the formulation of household products, this information was applied to both the industrial category, which covers where formulation takes place, and the consumer category, which would include product use. It was assumed that household/professional product composition would closely match the composition at the end of the industrial formulation process. This was seen in several documents related to environmental assessment [21, 22] where the same GFs were provided for both the process of industrial formulation and consumer use (SI-4).

## Results

The newer references supplemented previously identified sources and identified industrial GF information for multiple categories. Details and references are provided in SI-4 and summarized below. Industrial GF information for multiple product sectors was found on the ECHA Use Map website [18], although the level and placement of information varied by sector, and not all sectors included GF information. The documents do not have keywords or any mention of GF information in the titles; each was accessed and manually reviewed. Similarly, several OECD ESDs provided industrial pGF information [23–25], but not all the accessed documents did. Public portions of commercial websites were also manually searched and required multiple steps to drill down to exposure-relevant information, which included formulations for individual products, ingredients by product type, and ingredient functions [26, 27].

**Fig. 2:**
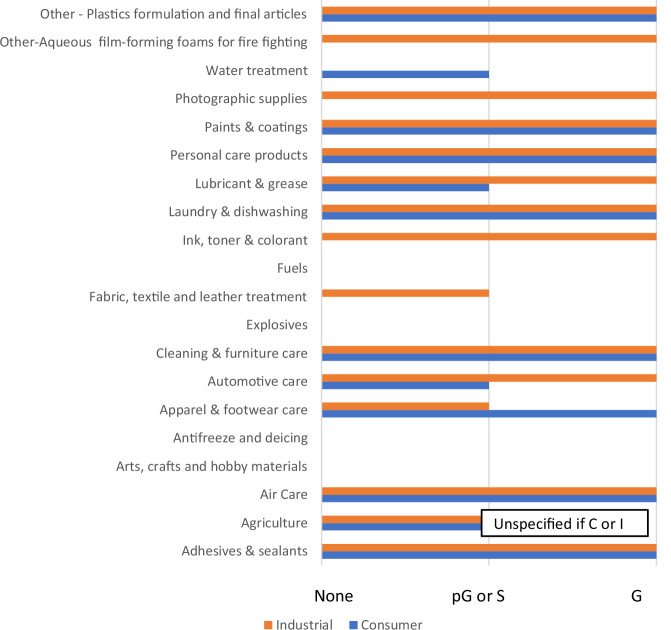
Summary of availability of Generic Formulations or other formulation-related Information by OECD Product Category from Reviewed References. The level of information identified is denoted as G (full generic formulation) or pG (partial generic formulation) or S (specific formulation information, which may or may not be representative of a product category). Results are shown by sector: Consumer (C), Industrial (I). I includes industrial, institutional and professional use products and formulation of household products (which would take place in an industrial setting). Finer distinctions can be found in SI-4. If information was provided for the process of formulation of household care products, this was assumed to apply to consumer as well.

**Fig. 3:**
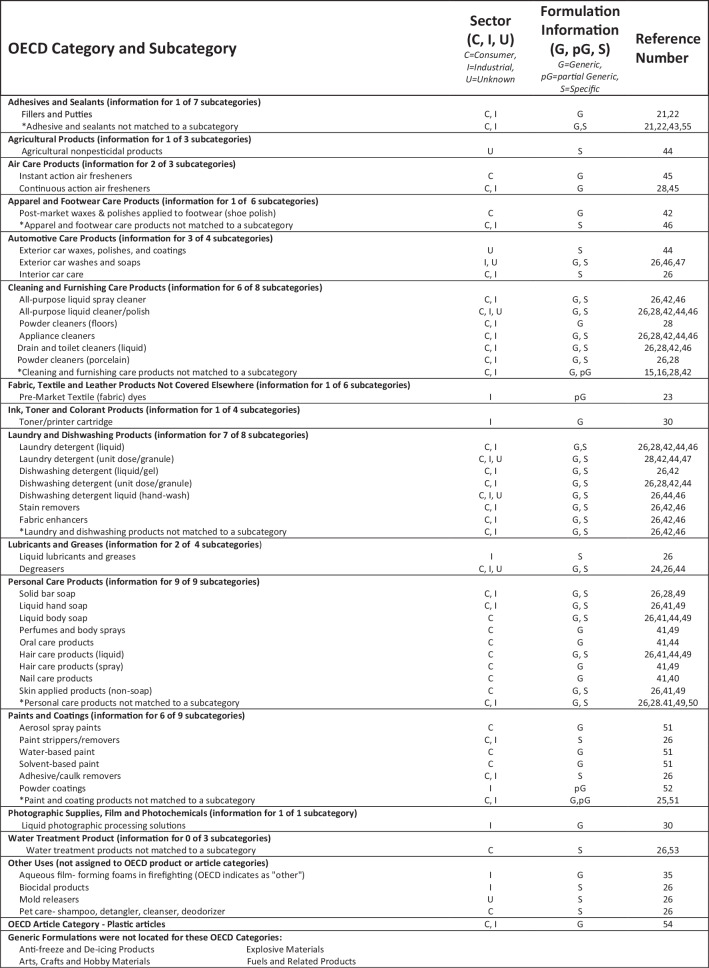
Mapping of generic formulation information and references to OECD Categories and Subcategories, to indicate information availability, the number of references found for each subcategory, and also the range of subcategories covered by a given reference. [15, 16, 21–26, 28–30, 40–55]. The level of information identified is denoted as G (full generic formulation) or pG (partial generic formulation) or S (specific formulation information, which may or may not be representative of a product category). Results are shown by sector: Consumer (C), Industrial (I). I includes industrial, institutional and also professional use products and formulations of household products (which would take place in an industrial setting). Finer distinctions can be found in SI-4. If information was provided for the process of formulation of household care products, this was assumed to apply to consumer as well.

A visual overview of the 20 use categories (the 18 OECD product categories, the one OECD “other” category that includes fire-fighting foams, and one OECD articles category of plastics formulation and final articles) is presented in Fig. 2 and a more detailed summary by subcategory in Fig. 3. Information was found for 16 of the 20 main use categories (Fig. 2) considering both consumer and industrial products. Fourteen categories had some level of industrial information, 11 categories had consumer information, and 1 category had information but was unspecified as to whether it was industrial or consumer. Most categories had multiple subcategories (Fig. 3 indicates information by subcategory). In some cases, matching information to a specific category-subcategory combination was not straightforward. In these situations, information was aligned with the main product category (Fig. 3, details in SI-4) rather than the subcategory. Figure 2 is based upon all information, i.e., mapped to a subcategory or main category.

In most cases, multiple subcategories were lacking information. Only the personal care product category had a clear match for each subcategory. The 19 OECD product categories (including “other”) represent a total of 91 subcategories, for which 36 had some level of GF information for consumer and/or industrial sectors, and 20 for industrial sectors. These counts do not include subcategories where only specific individual product information was identified. The level of GF information varied among sources, ranging from full formulations (product-based approach) to substance-specific weight fractions across product categories (substance-based approach) (Figs. 3 and SI-4).

We note that industrial formulations may be described in terminology challenging to align with OECD subcategories. As an example, formulations for “construction chemical products based on reactive epoxy resins (surface protection of concrete, concrete injection, waterproofing, floor screeds, flooring, functional coatings, adhesives for tiles)” [21] (SI-4) was not readily aligned with any of the adhesive subcategories listed in Fig. 3. In other cases, one GF was provided for products covering multiple subcategories [28] (SI-4).

This effort did not identify any GFs for four OECD categories: fuels; explosives; antifreeze and deicing; and arts, crafts and hobby materials. For the latter category, it is possible that some relevant information may be found in other categories (i.e., adhesives, coating, etc.), but without more specific descriptions, none could be assigned here. Previously identified consumer resources were included, but no specific effort was made to expand upon them.

Chemicals manufactured or imported in amounts equal to or greater than one million pounds per year comprise an estimated 95% of the total annual U.S. chemical production [29], and these substances are used to formulate both consumer and industrial products. But it is challenging to quantify what proportion of chemical production volume or commercial products the identified GF information represents. Our analysis describes the number of OECD categories and/or subcategories for which information was found, but a separate detailed analysis of commercial sales volumes of each would be needed to understand what overall market portion this represents. In addition, information was also identified for product types not readily assigned to OECD categories. Conditions of use may also need to be considered to understand if the information found covers products and constituents of greatest exposure potential and/or regulatory interest.

Overall, GF information was most often made available to support regulatory information needs or commercial marketing needs, with the exception of cleaning products. The latter seems to have been made available to proactively support product stewardship. Current sources of GFs for industrial or institutional processes include data to support registration of substances under REACH (Registration, Evaluation, Authorization, and Restriction of Chemicals) and documents released by specific manufacturers or formulators, sectors or companies (SI-4). There are no systematic guidelines across initiatives for making this information available. Nevertheless, it is to the benefit of the providers to ensure its accuracy because it is being made available to support regulatory or commercial activities.

Much information was found in sources that were not necessarily intuitive to search, and that may not be likely to be discovered with existing search strategies. For example, some industrial GFs were found in the I&P Europe Imaging and Printing Products Specific Worker Exposure Document (SWED) [30], which provides information for refining screening level defaults in occupational exposure modeling in the context of registration under REACH. The available information sources, however, did not contain an equal level of information on all addressed product types: GF information was not found in other SWED documents posted on ECHA’s Use Map website. Information available from ECHA’s Use Map website was examined for all sectors, and only those documents where useful information on the functional composition of products was found are included in the summary table.

While searching for GFs, other information types useful for exposure assessment, but historically data-limited, were also identified (included in SI-4). These include production or sales volume, sometimes by use categories; environmental release by media type; and daily use rates at industrial sites. Similarly to GFs, even within documents of the same type (OECD ESDs, REACH Specific Environmental Release Category (SpERC) background documents, etc.), this information differed in availability.

Manual queries of some commercial websites can identify raw materials and their functions for combinations of sector and product category. This type of information could inform the likelihood of presence in a product and potentially enable further development of QSURs.

## Discussion

### Industrial and institutional GF information availability for various commercial sectors

This current activity identified sources of formulation information for multiple sectors. In particular, the ECHA Use Map website [18] and OECD ESD documents, and sector or commercial websites were found to be useful resources for industrial and/or institutional formulation information. This information was often embedded within documents, spreadsheets, or websites. Resources were shared by co-authors from various sectors based on their personal knowledge. These observations suggest three points:

First, multi-stakeholder collaborative efforts are necessary to identify and use information available to support exposure assessment [1]. Indeed, this current activity resulted following a multi-stakeholder workshop where participants recognized sources of industrial formulation information were limited, leading meeting participants to identify further sources that could be explored.

Second, the relevant information in these newly identified resources was often hard to extract; that is, each document or resource type needed to be reviewed in detail to ensure proper extraction. The same level of detail is not found in similar documents, even within a given document type, such as OECD ESDs or SpERC Background Documents or Dutch National Institute for Public Health and the Environment (RIVM) ConsExpo Fact sheets, where the level of detail varied, and information placement varied- sometimes in the main text, sometimes in an annex. For some commercial websites, time-consuming queries are needed to pull up relevant information.

The third point is that without common, structured guidance, data may vary in type and quality across sources. Procedures for review and independent verification of each data source differ. The effort to develop this information and make it public is significant. Following common practices would make it easier to both identify this exposure-relevant information and increase confidence in its use. Examples of structured guidance for reporting exposure information that support data documentation, evaluation and findability include REACH guidance for use description and framework for exposure assessment and U.S. Environmental Protection Agency (EPA)Modeling Guidance [31–33]. A framework for further development of GF information should include the following:

- *Common format*. To best support exposure assessment, the development of a common format for consistent sharing of GFs would be helpful. International examples supporting harmonized data reporting include the OECD harmonization of use codes. Current publishing technologies are sufficiently developed to support standards such as machine-readable tables or online-archived open datasets.
- *Common guidance for parsing and assignment of data to categories and subcategories.* Development of common guidance would also need to address how to parse information between industrial and consumer situations. Appropriate parsing is needed to develop exposure scenarios representative of the range of use conditions and receptor populations. As discussed earlier, industrial information may cover manufacture, formulation, and use in industrial settings and/or use by professionals. In the latter case, formulations developed for industrial use may have the potential to be used by professionals in non-industrial settings. There is also potential for products made for trained professional use to end up in do-it-yourself markets. Additionally, information for the formulation of consumer products (an industrial activity) was considered to apply to the consumer products themselves. In such cases, while the composition remains similar, it should be recognized that environmental emissions likely have very different profiles (i.e., the difference between use at discrete industrial sites vs. widespread use and emission).
- *Development of a public database consistent with FAIR guidance.* A readily accessible GF database would be a valuable resource for exposure and risk estimation. A useful initiative to consider is the FAIR consortium [34]. Adhering to the FAIR guidelines would ensure that available data are accessible to their relevant audience and incorporated into analyses in a timely manner, reducing the need for extensive manual curation. This is critical to ensuring that GFs used to inform exposure assessment reflect emerging products, ingredients, and formulations.
  If more systematic and consistent creation and collation of GFs moves forward, there may be instances encountered for specialized product sectors where even this level of information may allow for partial inference of formulation strategies. Creative approaches could be tailored to ameliorate these instances and facilitate information availability- e.g., using broader concentration ranges, aggregation of functional categories, or other approaches as warranted.

### Considerations in GF application

The level of granularity in a GF may not be sufficient for some assessments. A functional category may include a broad range of chemical structures varying in hazard profiles, physical-chemical properties and environmental fate. It may also include complex substances (which contain multiple constituents based upon their derivation, e.g., gasoline distilled from crude oil, fragrances extracted from botanicals). For a substance with multiple sources and processes for production (e.g., plant-based and petroleum-based sources), understanding the impact of these on exposure throughout the lifecycle is currently beyond the scope of GF. The generic nature of GF supports its use in screening-level assessments, whereas more specific data are typically necessary for higher-tier assessments. In addition, because the GFs come from varying sources and are not based on consistent guidelines, users should assess them for their relevance and reliability for a given application.

### Additional useful information identified

REACH data provided a new source of information for industrial products or the formulation of industrial, professional, or consumer products. Beforehand, little information on formulation was known to be available for industrial situations. Information available for the industrial sector may have relevance for other sectors also. For example, products for industrial use can be differentiated into a) processing aids (e.g., metal part cleaners) and b) products fully or partly converted into articles. In the latter case, GFs, together with a basic understanding of function, can assist in predicting the composition of materials forming articles.

This review also found that public documents on the ECHA Use Map website provide additional data useful for exposure assessment (types of activities, conditions of use, environmental release rates, etc.). The availability of this information is noted in SI-4. Some, but not all, OECD ESD documents also include information on total production volume, sales volume by use categories, and release rates for some categories [25, 35]. The production volume and environmental release information are useful for estimating exposures of both environmental and human receptors.

### Potential future activities

- *Expansion of data search.* This paper describes a coordinated effort to identify and organize existing GFs for industrial products. Our analysis was not intended to be comprehensive, but to investigate whether some known sources could help fill information gaps for industrial formulations. There are several OECD categories and many OECD subcategories for which formulation data were not identified, which is also true of the consumer sector. A number of additional sources could be evaluated for further information (listed in SI-5). A comprehensive literature review or a detailed internet search strategy that uses artificial intelligence (AI) could also be designed, but would need to address the challenge identified here, that relevant online information may not be readily identifiable without prior knowledge. With sufficiently clear and practical terminology in the search request, one can get returns of chemical functions, representative chemistry delivering the function and concentration bands. Current AI technologies enable quick integration of many sources, delivering a starting point for further research or verification.
  Product databases provide opportunities for the development of empirical formulation information [36]. One example is the empirical function-based formulations for specific personal care product types developed by Isaacs et al. [6]. from chemical ingredient data derived from SDS. However, similar approaches for industrial products (e.g., formulations used in products and processes) have not been developed, primarily due to the lack of readily available data for specific products used in these contexts, especially compared to consumer data.
  Articles were beyond the scope of this activity, except for information from plastics master-batching found on the ECHA Use Map website. An additional and expanded effort to more carefully examine articles could be done. Residual concentrations of reactive substances in the unreacted form in cured products (resins, foams) or articles may be an important consideration for exposure assessment, but were beyond the scope of this information review.
  Additional developing resources may also be useful to examine as potential sources of formulation information. Where eco-labels exist, some level of GF information should also exist and may be accessible as a data source.
  Further analysis of the information identified here could also inform future efforts. Comparison of historical formulations with current ones may identify products with stable composition over time, as well as those more likely to change; the latter would be a greater priority to track over time. In addition, no extensive comparison of industrial vs. consumer formulations for a given product type was conducted. In a few cases, comparative observations are noted in SI-4 where the same resource provided information for both sectors. However, many consumer product companies devote considerable efforts to new product development and innovation, leading to an ever-changing landscape of proprietary formulations; but the extent to which these innovated products differ from GFs of the specific product category they reside in is not clear.
- *Future approaches for developing GFs.* Several ideas for expanding the availability of GF information will be discussed here, but underlying them all is the need to develop common terminology and structured templates for reporting information on GF. This type of activity is best designed in a multi-stakeholder setting, where both information providers and information users can provide input.
  Opportunities to explore how GFs could also be made available by other sectors can be examined. Considering the conditions (motivation, challenges, methods) under which the current data have emerged can help identify success factors for future application. Case studies that demonstrate the practical reliability and usefulness of available GF information may also be helpful.
  There may be potential to extrapolate information across GFs. For example, one reference [28] provided information for the formulation of household care and professional cleaning and hygiene products that covered multiple subcategories (SI-4). Extrapolation would require compositional knowledge of multiple product categories, and (because it is more general) may impact the resolution of the multicategory GF.
  Other approaches could be used to generate GF for products. These include the development of empirically derived GFs from large databases of product data curated from public documents (as were developed from the EU’s CosIng database for personal care products [7]). Databases such as the Consumer Product Information Database and the EPA’s Chemicals and Products Database (CPDat) provide large amounts of consumer product data for expanding empirical formulations to other types of products [9, 37]. There is an ongoing effort to expand CPDat to occupational and industrial formulations, increasing the available data for developing formulations for this sector to augment the sources reported herein [38]. Other approaches include the use of NTA to develop measurement-based recipes for consumer products [39], although current NTA methods are limited in their ability to generate quantitative information.
- *Incorporation into Technology-Enabled Methods for Exposure and Safety Assessment.* Standardization of GF information into a machine-readable format would enable the application of automated AI-driven tools to extract, interpret, and apply information across product databases. This could support scalable exposure scenario generation, automated input population for predictive exposure tools and improved integration with QSUR models to infer concentration and function.
- *Expansion of OECD categories.* Product descriptions were sometimes not readily assignable to OECD product categories. Further description of the categories may aid in data parsing and category assignment, or additional subcategories may be useful. Also, some functional categories may contain a broad range of chemistries or complex substances. A combination of function and chemical class may be an option to extend GF utility.

### Significance

This paper highlights available GF data, advocates for a more systematic framework to organize and obtain these data and explains the benefits of having this information available for various user sectors.

The GF information identified here can be used to:

- support chemical exposure, safety, and/or risk assessment. GF information supports qualitative and quantitative assessment as it can be used to identify the likelihood of presence and approximate concentration within a formulated product.
- share knowledge with the public sector interested in having a better understanding of chemical use.
- benchmark concentration predictions from QSURs as they expand to the prediction of quantitative data for functions of substances in products.
- expand the domain of applicability of QSURs, particularly to the industrial/formulation area. Sources that provide ingredients by both name and function for a given product category (i.e., [26, 27]) can be used to further develop or benchmark existing QSUR predictions of function.
- understand functional components integral to product types, which can help with new or alternate formulations and sustainable product design.

In addition, while searching references for GF information, other types of exposure-relevant information were identified (e.g., environmental release rates, production or sales volume by market, daily use rates at industrial sites). This information can be applied to benchmark or refine environmental emission inputs and predictions. It should be noted that this information is specific to the manufacturing processes and raw material sources described and may not cover emissions for all process- or source-variations for a given substance.

The GF sources identified in this effort indicate greater availability of industrial formulation information than previously recognized, yet also highlight the limited information available given the range of products in commerce. It should again be noted that this effort was intended to explore the availability of GF information for industrial chemical products, and it was by no means a comprehensive review for industrial or consumer information. Future efforts to expand the availability of GF information would be useful. These efforts, if undertaken, should include all stakeholders. Sector organizations generally have access to the information needed to make available GFs by functional ingredients. Compilation and aggregation could be supported by other types of third parties acting as honest brokers of information. A third-party approach may also enhance the credibility of non-peer-reviewed information. Data would be most useful if compiled in a structured, harmonized format to support data identification, searchability and utilization.

## Supplementary information

Supplementary Information-1,2,3,5

Supplementary Information-4

## Data Availability

Analysis based upon public sources of information and data cited within the document.
