# Supplementary material for: Generic formulations: availability and applicability for exposure assessment
Source: J Expo Sci Environ Epidemiol. 2026 Mar 16;36(3):479–89. doi: 10.1038/s41370-025-00837-4 (PMC13143825; doi:10.1038/s41370-025-00837-4)
Supplement: Supplementary file 1 — Supplementary Information-1,2,3,5 [file 41370_2025_837_MOESM1_ESM.pdf]

Supplementary Information Sections 1,2,3 and 5

SI-1: Additional discussion of how use-related information informs chemical risk assessment p. 1-2

SI-2: Example of an Application of a GF p. 3-5

SI-3: Terminology p. 6-7

SI-5: Potential sources of GF-relevant information p. 8-9

Supplementary Information 1:
Additional discussion of how use-related information informs chemical risk assessment

Conditions of use, including quantitative chemical concentrations, compartments of release, and end-user populations are relevant to exposure estimation via different sources, scenarios, and pathways. Here, we provide some practical examples of the importance of such data in exposure and other related assessments. Key use-related parameters for exposure and risk assessment include what types of products and processes substances are used in, concentration levels within these products and processes, and environmental emissions associated with manufacture and/or product use. We provide a brief introduction to each of these parameters here, to further support the main text discussion of how generic formulation and other information identified in this effort relate to each of these parameters.

The information on the product and process types in which a substance is likely to appear plays a key role in exposure and safety assessment. Product types are mostly associated with certain user groups and use conditions, from which critical exposure potential and related risk management needs can be deduced. In addition, for reactive substances (e.g., substances that undergo 'rapid' chemical transformations into other substances), it is consequential for manufacturers and regulators to understand at which point in the supply chain the concentration of the substance decreases to a level of no concern. This is particularly important where formulations or articles are foreseen to be used by consumers or untrained professionals.

Once it is known in which products a substance is likely to be present, another key parameter is the concentration of the substance in the product /process application. Models for predictive estimation of exposure from dermal or oral contact to consumer products often require this input exposure determinant. Also, for endpoints where thresholds apply (toxicological or regulatory), the concentration drives the risk. In many cases, however, banding of concentration in orders of magnitude may be sufficient and a precise value may not be needed, especially at a screening level. For substances present in articles, the significance of concentration as an exposure driver often depends heavily on physicochemical properties of the substance and the diffusivity of the material matrix, such that concentration can matter much more for small molecules in polypropylene/polyethylene (PP/PE) matrices than for large molecules (e.g. > 1500 Da) in less diffusive matrices like polyesters. In addition to exposures associated with direct product use, low-level chronic exposures may occur for substances that persist in the environment.

For occupational exposure estimates, the concentration of a chemical used in industrial formulations or in specific processes can drive exposure, particularly in open applications. The significance again

depends on the intrinsic potential of the substance to become airborne (fugacity), the amount of energy introduced during the work process, and the potential interaction with the material matrix. The substance concentration in products is also relevant to estimation of indoor air exposure arising from building material and interior article sources. The concentration in single product types together with market data on these product types is key to calculate the overall indoor presence of a substance.

Alternatively, for environmental exposure estimation, the amount of substance released into various environmental compartments is the key driver of exposure (similar to indoor air but with a higher diversity of potential sources), whether it be release from industrial facilities during manufacture, formulation or use, or residential out-the-window or down-the-drain release during use. Concentration may matter when calculating/testing releases from products during use, but most environmental models work with standard emission factors applied to a fraction of total market tonnage supplied into a certain (sub)sector or product group, to which then a generic emission factor applies. The concentration may mostly matter for industrial users of mixtures for calculating their potential release into environment, (and thus for informing priorities for their environmental risk management on-site- e.g., substances in textile and paper finishing or metal processing chemicals). The emission factor may vary, for example, driven by source of raw material, manufacturing technique and/or pollution control measures.

Other exposure factors beyond the scope of this paper, such as worker Standard Operating Practices or consumer habits and practices – i.e., behaviours related to contact with products and articles, are also important considerations in exposure assessment. It is important to note that all factors should be considered when defining the scope of an exposure assessment. Only then can an evaluator recognize, select, and focus on the most pertinent factors.

Supplementary Information 2:

Example of an Application of a GF

The following is one example of how GF information can inform an exposure assessment. It follows the chemical specific path of Figure 1 in the text.

Problem Formulation:

Chemical A is present in a cleaning product as a colorant. What levels of Chemical A may an individual using the product be exposed to?

Approach: This is an illustrative example, so a hypothetical chemical, Chemical A is used. Physical chemical properties are set at nominal values, MW=100, VP = 100 torr, log Kow = 2. USEPA's CEM model, an exposure model that includes a scenario for use of a liquid general purpose cleaner, is used. The conditions of use are left at model defaults (while these represent consumer household use, not an industrial setting, the purpose here is to demonstrate the relative impact of adjusting product information based upon GFs).

A) Exposure prediction without consulting GF:

Physical-chemical property information are entered into the model. The CEM uses a default weight fraction for the scenario of 0.5 (or 50%). The model predicts acute dose rates of 48 mg/kg/day for inhalation and 34 mg/kg/day for dermal routes.

Exposure prediction applying GF information:

A generic formulation is available for liquid cleaning products:

Table 1. Generic Formulation for liquid cleaning products, low viscosity (based upon AISE 2021b, which contains these and additional functional categories).

Function	Range in product (%)
Abrasive	0-10
Alkalinity Source	0-10
Builders	0-15
Chelants	0-0.2
Color agents/dyes	0-1
Enzymes	0-1
Fragrances	0-5
Preservatives	0-1.5
Solvents	0-50
Surfactants	0-30
Viscosity control	0-4

The weight fraction in the model is adjusted to the upper bound for color agents/dyes specified in the GF, 0.01 or 1%, and the model is run with the same conditions of use. Table 2 contains results for both model runs.

Table 2. Exposure Predictions for Chemical A for Adult User of Liquid Cleaning Product by exposure route. A comparison of estimates based upon model default weight fraction (which equals the highest weight fraction of any functional category in the GF) and the GF weight fraction for Chemical A based upon its function within the product.

Model Run	Inhalation Acute dose rate (mg/kg/day)	Dermal Acute dose rate (mg/kg/day)
Default (0.5 WF)	48	34
WF adjusted based upon GF – colorant specific value (0.01 WF)	1	0.7

Possible approaches when function is not known:

Application of Quantitative Structural Use Relationships (QSURs): For this example, the function of Chemical A was known. If it was not available, USEPA has developed QSURs to predict function based upon a substance's chemical structure. These predictions are currently limited to consumer products. QSURs could be developed for industrial products if a sufficient public dataset was available to support their development.

Consult references that provide functions for chemicals in products (i.e. Stepan, UL Prospector, ACI, other sources in SI-3 or SI-5 potential information sources).

Apply highest concentration category from GF: We note that some exposure models do not have defaults for weight fractions but require user entry. In that case, the concentration information in the GF can be integral to the assessment. If the ingredient function was not known, an upper bound could be estimated based upon the concentration range for the functional constituent with the highest range, or the highest percentage of unaccounted functional constituents, whichever may be higher.

Possible approaches when physical-chemical properties are not known: Several exposure models use a banding approach, where exposure levels are associated with ranges of physical-chemical properties (ECETOC TRA worker and consumer modules, EGRET for consumer are examples). Here the physicochemical property bands that predict the highest exposure concentrations could be used for screening level exposure estimates.

References:

American Cleaning Institute. Cleaning Chemistry Catalog https://www.cleaninginstitute.org/industry-priorities/science/cleaning-chemistry-catalog. Accessed 17 August 2025.

European Center for Ecotoxicology and Toxicology of Chemicals (ECETOC) Targeted Risk Assessment (TRA): https://www.ecetoc.org/tools/tra-main/. Accessed 16 August 2025

European Solvent Industry Group Generic exposure scenario Risk and Exposure Tool (EGRET): https://www.esig.org/ges/consumers/ Accessed 16 August 2025.

Stepan. Products and Markets Formulation Finder. 2024. https://www.stepan.com/content/stepan-dot-com/en/products-markets/formulation-finders/formulations.html. Accessed 29 June 2024.

ULTRUS Prospector. Ingredient Search & Raw Materials Search Engine. https://www.ulprospector.com/en/na. Accessed 29 June 2024.

USEPA CEM model: https://www.epa.gov/tsca-screening-tools/cem-consumer-exposure-model-download-and-install-instructions. Accessed 16 August 2025.

SI3:

Terminology

Articles are:

OECD: “generally solids, polymers, foams, metals, woods, which are always present…for the duration of their useful life which may be several years.” (OECD 2025).

Registration, Evaluation and Authorization of Chemicals (REACH) Regulation: defines articles as “an object which during production is given a special shape, surface or design which determines its function to a greater degree than its chemical composition” (EC 2006).)

USEPA Toxic Substances Control Act (TSCA) definition: “Article means a manufactured item (1) which is formed to a specific shape or design during manufacture, (2) which has end use function(s) dependent in whole or in part upon its shape or design during end use, and (3) which has either no change of chemical composition during its end use or only those changes of composition which have no commercial purpose separate from that of the article, and that result from a chemical reaction that occurs upon end use of other chemical substances, mixtures, or articles”[CFR].

Safety Data Sheets: communicate information on hazard, occupational safety and health and environmental safety. There is a standard specification for types of content UN Globally Harmonized System of Classification and Labelling of Chemicals (Wikipedia, accessed 8/13/2025) https://en.wikipedia.org/wiki/Safety_data_sheet

ECHA’s use map site: https://www.echa.europa.eu/csr-es-roadmap/use-maps/use-maps-library site contains a library of information on use and conditions of use. This information is typically developed by downstream user sector organizations.

FEICA: the Association of the European Adhesive and Sealant Industry, one of the use sector organizations that developed information found on ECHA’s use map site.

EFCC: the European Federation for Construction Chemicals, one of the use sector organizations that developed information found on ECHA’s use map site.

Industrial products: for the purposes of this document, products marketed primarily for use at industrial or professional workplaces, rather than residential use by the general population.

Products are:

OECD : “consumable liquids, aerosols, semi-solids, or solids that are used a given number of times before they are depleted” [OECD 2025].

REACH: Formulated product = mixture = means a mixture or solution composed of two or more substances [EC 2006]

References:

CFR Regulation. 40CFR 704.3. 15 USC Ch. 53: TOXIC SUBSTANCES CONTROL. https://uscode.house.gov/view.xhtml?path=/prelim@title15/chapter53&edition=prelim.

Organization for Economic Co-operation and Development (OECD). Internationally harmonised functional, product and article use categories, Second edition. 2025. OECD Publishing, Paris. https://doi.org/10.1787/2e7db924-en

European Commission (EC). Regulation (EC) No 1907/2006 of the European parliament and of the council. 2006. https://eur-lex.europa.eu/legal-content/en/TXT/HTML/?uri=CELEX:02006R1907-20231201.

Supplementary Information 5:

Potential sources of GF-relevant information

This document demonstrates the availability of generic formulation (GF) information for industrial products based upon sources known to the authors. In addition to the sources reviewed for this paper, other potential sources of GF for primarily consumer products were identified during the process of manuscript development. They are briefly mentioned in the paper but included here in more detail so that they can be considered by any future efforts to systematically capture GF information.

Potential sources to consider in future include:

Information Source	Type of Information	Availability
ASTM and ISO Standards	Standards that discuss how to measure content, migration or emission from products or materials and often include discussion of formulations.	These must be purchased, links to access these and other standards are found on the Intertek site listed below.
Flick's Formulations	Publicly available books with consumer product formulation data for several use categories. Include Paint and Ink Formulations Database (2005), Water-Based Paint Formulations (1995), Cosmetics and Toiletry Formulations (2007), Advanced Cleaning Product Formulations (1994)	Must be purchased
Labeling initiatives such as Labeling of Hazardous Materials in Art Act (LHAMA) and Eco-Labeling Initiatives	Require testing and labeling, include information on composition and constituents. Public availability may vary.	Example: Art Materials | CPSC.gov:
Art and Craft Safety Guide (USCPSC),	Indicates some constituents and associated hazards of arts and crafts products	blk_media_5015-
Patents	Patents often include quantitative information on how much of the chemical substance is needed to perform a function as well as categorical information about use descriptors. Examples of how patent data were incorporated into recent CPSC reports can be found https://www.cpsc.gov/s3fs-public/Vol%201%20Main%20Report%2003.24.2022_Final_with%20cover.pdf?VersionId=y70jyWfP5ROcTlb5Kon40Ht1QgXkFl3m	millions of searchable patents on thousands of chemical substances on PUBCHEM https://pubchem.ncbi.nlm.nih.gov/
Sites that provide functional categories for product constituents	Function information can help understand presence when linked with GF	Examples include: Cleaning Chemistry Catalog: https://www.cleaninginstitute.org/industry-priorities/science/cleaning-chemistry-catalog Consumer Product Ingredients Database: https://productingredients.com/

High Priority Chemicals Data System (HPCDS)	Include information on chemicals in children's products as required by the Oregon Toxic-Free Kids Act, the Washington Children's Safe Products Act and the Vermont Chemical Disclosure Program and priority chemicals in general consumer and commercial products for Safer Products for Washington state	https://hpcds.theic2.org/search
Consumer Product Standards	May include info on constituents and composition. May be fees to access.	The Intertek website https://www.intertek.com/testing/consumer-products/ provides a link to Search and Access Consumer Product Standards https://www.intertekinform.com/en-us/key_standards/consumer_product_standards/
Programs and resources that support product labeling programs	Have information on composition and constituents, but public access may be limited.	Examples include:<br>Duke Toxicology Program: https://fmch.duke.edu/divisions/division-occupational-environmental-medicine/duke-toxicology-program<br>Eurofins: https://www.eurofinsus.com/consumer-product-testing/<br>UL Research Institutes: https://ul.org/institutes-offices/chemical-insights/
References on emissions from products	Give indications of constituents	Example: https://doi.org/10.1016/j.envint.2023.108316
Chemical Data Reporting Database	Reporting now (2024 onward) aligns with OECD use categories	https://www.epa.gov/chemical-data-reporting/access-chemical-data-reporting-data
Product databases to support poison centers	Include information on product and substance names, composition and use	More information can be found in: doi.org/10.1016/j.taap.2005.02.026
RIVM Consumer Product Fact Sheets	Primarily for consumer products. Individual ones were included in this review	https://www.rivm.nl/en/consexpo/fact-sheets
Cosmetics Products Notification Portal	Information for cosmetics products	https://single-market-economy.ec.europa.eu/sectors/cosmetics/cosmetic-product-notification-portal_en.
